# Editorial: Transcription and chromatin regulators in neurodevelopmental disorders

**DOI:** 10.3389/fnins.2022.1023580

**Published:** 2022-09-16

**Authors:** Debbie L. C. van den Berg, Julian Ik-Tsen Heng, Alessandro Sessa, Cristina Dias

**Affiliations:** ^1^Department of Cell Biology, Erasmus University Medical Center, Rotterdam, Netherlands; ^2^Remotely Consulting, Perth, WA, Australia; ^3^Division of Neuroscience, IRCCS San Raffaele Scientific Institute, Milan, Italy; ^4^Department of Medical and Molecular Genetics, Faculty of Life Sciences and Medicine, King's College London, London, United Kingdom; ^5^Neural Stem Cell Biology Laboratory, The Francis Crick Institute, London, United Kingdom

**Keywords:** neurodevelopmental disorders, chromatin, gene regulation, differentiation, brain, neurodevelopment, transcription

Disruptions to key cellular processes during mammalian brain formation such as cell proliferation, lineage-specification and differentiation (Miller et al., 2019) lead to neurodevelopmental disorders (NDDs) in humans (Ross and Walsh, 2001; Subramanian et al., 2019). Among many signaling mechanisms underlying these processes, the functions of transcription and chromatin regulators of gene expression arguably represent the most prominent (Oproescu et al., 2021; Bölicke and Albert, 2022; Fujita et al., 2022). This topic explores how their defective function leads to NDDs, and how clarifying genotype-phenotype relationships of NDDs facilitates delineation of unique pathological traits.

Reichard and Zimmer-Bensch overview the molecular regulation of brain development, focusing on the role of chromatin modifiers, as well as consequences flowing from their functional impairment. The authors discuss how environmental stressors such as malnutrition or mental/neurophysiological stress during pregnancy triggers epigenetic changes that affect brain and behavior. Along this theme, Sokpor et al. discuss cell delamination during cortical development, an indispensable process for tissue neurogenesis, gliogenesis and tissue growth. The authors discuss how transcription and chromatin regulators mediate cellular detachment from germinal zones of the embryonic cortex, as well as how disruptions to such processes are connected with NDDs.

Overlapping clinical features related to both physical appearance and cognitive function are described across NDDs, yet the mechanistic basis for these shared NDD traits remains poorly understood. Larizza et al. investigate Rubinstein-Taybi syndrome (RSTS) and Heterogeneous Nuclear Ribonucleoprotein H1 (hnRNPH1)-related syndromic intellectual disability (ID) to describe a putative chromatin and transcriptional regulatory network underpinning shared phenotypes. RSTS is frequently caused by monoallelic pathogenic variants in genes encoding CREB-binding protein (CBP) as well as EP300, homologous lysine acetyltransferases that mediate histone H3 lysine 27 acetylation at enhancers (Hennekam, 2006; Raisner et al., 2018). The authors show that alternative pre-mRNA splicing regulators hnRNPs (including hnRNPH1) are strongly downregulated in RSTS iPSC-derived neurons, and CBP/EP300 could also acetylate hnRNPs to modulate their signaling. Thus, misregulated RNA processing could represent a common underlying mechanism for clinical similarities observed across HNRNPH1-related syndromes and RSTS.

In keeping with Larizza et al.'s hypothesis of converging mechanisms underlying phenotypic similarities in chromatin disorders, Parenti and Kaiser review Cornelia de Lange Syndrome (CdLS) as a phenotypic spectrum of transcriptional regulation and chromatin remodeling. CdLS diagnoses are largely attributable to mutations in *NIPBL*, a gene encoding the cohesin loading factor Nipped-B-like protein (NIPBL) (Tonkin et al., 2004). Functional assays on patient cells show that the underlying mechanism for disease-causing mutations may not lie solely in its sister chromatid cohesion function. Rather, evidence suggests broader roles in transcriptional regulation, RNA polymerase II (RNApol2) interaction and long-range chromatin contacts, collectively relevant to cellular homeostasis and disease. Furthermore, disease-associated variants in *BRD4*, encoding a histone acetyl lysine “reader” and interactor of NIPBL (Olley et al., 2018), may cause CdLS-like conditions through disruption of NIPBL function. Correspondingly, variants in NIPBL-interacting transcription factors and chromatin regulators are associated with classical CdLS and CdLS-like features that are also shared with other NDDs. Eigenhuis et al. further explore CdLS heterogenous etiology within the context of genes encoding transcriptional pause-release regulators. Notably, TFIID functions as a General Transcription Factor (GTF) in the preinitiation complex (PIC), an essential component for gene transcription, pausing and release (Schier and Taatjes, 2020). Multiple components of its TBP-associated factors (TAFs) interact with the superelongation complex (SEC), including BRD4 and SEC that associate with positive transcription elongation factor-b (P-TEFb) complex, which is then recruited to paused RNApol2, leading to efficient transcription elongation. Mutations to transcriptional pausing regulators cause NDDs with craniofacial and limb anomalies often reminiscent of a CdLS phenotype. A phenotype-to-function hypothesis is presented that reconciles genetic mutations to possible and plausible pausing regulators (including *KMT2A* and *ANKRD11*) to defective transcription and cellular dysfunction, leading to disease.

Further exploring chromatin regulators in NDDs reminiscent of CdLS and RSTS, Vasko and Vergano study Coffin-Siris Syndrome (CSS), a condition arising predominantly from mutations to the SWI/SNF chromatin remodeling complex. In a large cohort of CSS cases, the authors report mutations in genes encoding SWI/SNF proteins (most commonly ARID1B) and associated transcription factors (SOX11, SOX4), and explore potential genotype-phenotype associations with features including language deficits and adaptive interventions.

Different mutations to a single gene can also precipitate multiple NDDs. Antonyan and Ernst explore how stabilizing and heterozygous loss-of-function mutations in SET binding protein 1 (SETBP1) affect brain development to cause Schinzel-Giedion syndrome (SGS) or SETBP1 deficiency disorder (SDD), respectively. The authors describe how SETBP1 signals through SET which, together with TAF1A and ANP32A, forms the INHAT (inhibitor of acetyltransferase) complex to negatively regulate histone acetyltransferases such as CBP/EP300 (Seo et al., 2001). Other mechanisms, including regulation of AKT signaling, DNA repair, and cell cycle control are also considered.

Rett syndrome (RTT) is a severe neurological condition affecting almost exclusively females and largely caused by mutations in the *X*-linked gene *MECP2* (Lyst and Bird, 2015). The gene encodes methyl-CpG-binding protein 2, an essential gene expression regulator (Tillotson and Bird, 2019) that is abundant in neuronal cells and relevant to their morphology (Shahbazian et al., 2002). Yet, its role in astrocytes remains understudied in RTT (Lioy et al., 2011; Garg et al., 2015). Albizzati et al. address this by quantifying brain astrocyte heterogeneity in an RTT mouse model (Burda and Sofroniew, 2014). They found *Mecp2* null astrocytes were morphologically less complex compared to wildtype, and such differences were regionally variable in the brain, particularly within motor and somatosensory cortices. Thus, *Mecp2* may mediate astrocyte morphology, possibly through chromatin remodeling in RTT.

In conclusion, these studies collectively illustrate the wide-ranging roles for transcription and chromatin regulators in neurodevelopment. Taken alongside current advancements in gene editing, improved pharmacological interventions that modulate such signaling in cells, as well as innovative functional assays to interpret the impact of causative vs. benign genetic variants; we collectively move closer to the promise of precision diagnoses and personalized treatments for NDDs in humans.

## Author contributions

All authors contributed to writing and approved the submitted version.

## Funding

CD is supported by the Wellcome Trust (209568/Z/17/Z). AS is supported by the Italian Ministry of Health (GR-2019- 12370949), and Fondazione Regionale per la Ricerca Biomedica (Regione Lombardia), European Joint Programme on Rare Disease Project TREAT-SGS (EJPRD20-008), GA 825575.

## Conflict of interest

JH is Founder and Director of Remotely Consulting and did not receive salary for this work. The remaining authors declare that the research was conducted in the absence of any commercial or financial relationships that could be construed as a potential conflict of interest.

## Publisher's note

All claims expressed in this article are solely those of the authors and do not necessarily represent those of their affiliated organizations, or those of the publisher, the editors and the reviewers. Any product that may be evaluated in this article, or claim that may be made by its manufacturer, is not guaranteed or endorsed by the publisher.
